# Child Adult Relationship Enhancement in Primary Care (PriCARE): study design/protocol for a randomized trial of a primary care-based group parenting intervention to prevent child maltreatment

**DOI:** 10.1186/s13063-022-07024-y

**Published:** 2023-02-23

**Authors:** Samantha Schilling, Byron J. Powell, Paul W. Stewart, Joanne N. Wood

**Affiliations:** 1 Department of Pediatrics, School of Medicine, The University of North Carolina at Chapel Hill, Chapel Hill, USA; 2 Center for Mental Health Services Research, Brown School, Washington University in St. Louis, St. Louis, USA; 3 Center for Dissemination & Implementation, Institute for Public Health, Washington University in St. Louis, St. Louis, USA; 4 Division of Infectious Diseases, John T. Milliken Department of Medicine, School of Medicine, Washington University in St. Louis, St. Louis, USA; 5 Department of Biostatistics, Gilling School of Global Public Health, The University of North Carolina at Chapel Hill, Chapel Hill, USA; 6 Safe Place: The Center for Child Protection and Health, PolicyLab, Center for Pediatric Effectiveness and Division of General Pediatrics, Children’s Hospital of Philadelphia, Philadelphia, USA; 7 Department of Pediatrics, Perelman School of Medicine at the University of Pennsylvania, Philadelphia, USA

**Keywords:** Positive parenting intervention, Primary care, Child maltreatment prevention, Behavior problems, Implementation determinants, Hybrid 1 effectiveness-implementation trial

## Abstract

**Background:**

Child maltreatment (CM) is a pervasive public health problem and there is a critical need for brief, effective, scalable prevention programs. Problematic parent-child relationships lie at the heart of CM. Parents who maltreat their children are more likely to have punitive parenting styles characterized by high rates of negative interaction and ineffective discipline strategies with over-reliance on punishment. Thus, parenting interventions that strengthen parent-child relationships, teach positive discipline techniques, decrease harsh parenting, and decrease child behavioral problems hold promise as CM prevention strategies. Challenges in engaging parents, particularly low-income and minority parents, and a lack of knowledge regarding effective implementation strategies, however, have greatly limited the reach and impact of parenting interventions. Child Adult Relationship Enhancement in Primary Care (PriCARE)/*Criando Niños con CARIÑO* is a 6-session group parenting intervention that holds promise in addressing these challenges because PriCARE/*CARIÑO* was (1) developed and iteratively adapted with input from racially and ethnically diverse families, including low-income families and (2) designed specifically for implementation in primary care with inclusion of strategies to align with usual care workflow to increase uptake and retention.

**Methods:**

This study is a multicenter randomized controlled trial with two parallel arms. Children, 2–6 years old with Medicaid/CHIP/no insurance, and their English- and Spanish-speaking caregivers recruited from pediatric primary care clinics in Philadelphia and North Carolina will be enrolled. Caregivers assigned to the intervention regimen will attend PriCARE/*CARIÑO* and receive usual care. Caregivers assigned to the control regimen will receive usual care only. The primary outcome is occurrence of an investigation for CM by child protective services during the 48 months following completion of the intervention. In addition, scores for CM risk, child behavior problems, harsh and neglectful parenting behaviors, caregiver stress, and caregiver-child interactions will be assessed as secondary outcome measures and for investigation of possible mechanisms of intervention-induced change. We will also identify PriCARE/*CARIÑO* implementation factors that may be barriers and facilitators to intervention referrals, enrollment, and attendance.

**Discussion:**

By evaluating proximal outcomes in addition to the distal outcome of CM, this study, the largest CM prevention trial with individual randomization, will help elucidate mechanisms of change and advance the science of CM prevention. This study will also gather critical information on factors influencing successful implementation and how to optimize intervention referrals, enrollment, and attendance to inform future dissemination and practical applications.

**Trial registration:**

This trial was registered on ClinicalTrials.gov (NCT05233150) on February 1, 2022, prior to enrolling subjects.

## Administrative information

Note: the numbers in curly brackets refer to SPIRIT checklist item numbers (https://www.spirit-statement.org/interventions). The order of the items has been modified to group similar items (see http://www.equator-network.org/reporting-guidelines/spirit-2013-statement-defining-standard-protocol-items-for-clinical-trials/).

**Table:** 

Title {1}	Child Adult Relationship Enhancement in Primary Care (PriCARE): study design/protocol for a randomized trial of a primary care-based group parenting intervention to prevent child maltreatment
Trial registration {2a and 2b}.	ClinicalTrials.gov: NCT05233150
Protocol version {3}	31 October 2022, version 1.0
Funding {4}	NICHD (1R01HD103902-01)
Author details {5a}	^1^Department of Pediatrics, School of Medicine, The University of North Carolina at Chapel Hill, Chapel Hill, USA ^2^Center for Mental Health Services Research, Brown School, Washington University in St. Louis, St. Louis, USA ^3^Center for Dissemination & Implementation, Institute for Public Health, Washington University in St. Louis, St. Louis, USA ^4^Division of Infectious Diseases, John T. Milliken Department of Medicine, School of Medicine, Washington University in St. Louis, St. Louis, USA ^5^Department of Biostatistics, Gilling School of Global Public Health, The University of North Carolina at Chapel Hill, Chapel Hill, USA ^6^Safe Place: The Center for Child Protection and Health, PolicyLab, Center for Pediatric Effectiveness and Division of General Pediatrics, Children's Hospital of Philadelphia, Philadelphia, USA ^7^Department of Pediatrics, Perelman School of Medicine at the University of Pennsylvania, Philadelphia, USA
Name and contact information for the trial sponsor {5b}	Corresponding Author: Samantha Schilling, Department of Pediatrics, UNC School of Medicine, 231 MacNider Hall, Chapel Hill, NC 27599, US; samantha_schilling@med.unc.edu; phone: 919-966-2504; fax: 984-974-0875
Role of funder{5c}	The funder had no role in the design of this study and will not have any role in its execution and publication.

## Introduction

### Background and rationale {6a}

Child maltreatment (CM) is a pervasive public health problem: an estimated 37% of all US children are investigated by Child Protective Services (CPS) for suspected abuse or neglect before their 18th birthday [1]. Maltreated children are at increased risk for lifelong adverse health, social, and economic consequences including mental health problems [2, 3], chronic disease [4, 5], disability [6], delinquency and criminality [7, 8], and decreased economic productivity [9]. Given the high prevalence and substantial consequences of CM, the societal costs are enormous: the annual US economic burden of CM is estimated to be $2 trillion [10].

CM results from a complex interaction of child, parent, and environmental factors [11–13]. Young age and behavior problems are key child-level risk factors for CM. In fact, the vast majority of maltreatment-related hospitalizations and fatalities occur in children under the age of 6 years [14, 15]. Child behavior problems are both a risk factor for triggering CM and a consequence of abuse and/or neglect [16]. Parent-related factors including poor parenting skills, mental health problems, and drug/alcohol abuse also have a strong influential role in CM [11, 17]. Furthermore, parents who experienced CM are more likely to maltreat their own children [18, 19]. Low socioeconomic status (SES) is the strongest and most consistent environmental predictor of CM [20]. Children in low SES households are 3 times more likely to be abused and 7 times more likely to be neglected than those in higher SES households [21].

Positive parenting interventions are a promising CM prevention strategy. Both physical abuse and neglect represent extreme forms of problematic parenting [16]. Parents who maltreat their children are more likely to use ineffective discipline with an over-reliance on punishment [22, 23]. Harsh, reactive parenting can contribute to the development of child behavior problems, which can increase parental stress and escalate negative parenting behaviors, including abusive and neglectful parenting [24]. Parenting interventions that strengthen the parent-child relationship, teach positive discipline techniques, and promote authoritative parenting have been identified as key evidence-based CM prevention strategies by the National Academy of Medicine (NAM) and others [16, 25–28]. Grounded in attachment and social learning theory [29, 30], these programs aim to (1) improve parent-child interactions, (2) increase understanding of normal child development, (3) promote understanding of the negative impact of attention to problem behavior and lack of attention to positive behavior, and (4) teach positive discipline practices. By promoting the use of positive parenting skills and decreasing harsh parenting, parenting programs are effective in reducing child behavior problems [27] which are a risk factor for CM [16]. Thus, parenting programs have the potential to reduce risk of CM through their direct impact on parenting behaviors and through the impact of those changes in parenting behaviors on child behaviors. Research demonstrates that skills-based group positive parenting programs improve several proximal factors related to CM, including family functioning, parent stress, and child behavioral problems, yet the impact of these programs on CM remains unknown [27, 31–35].

**Fig. 1:**
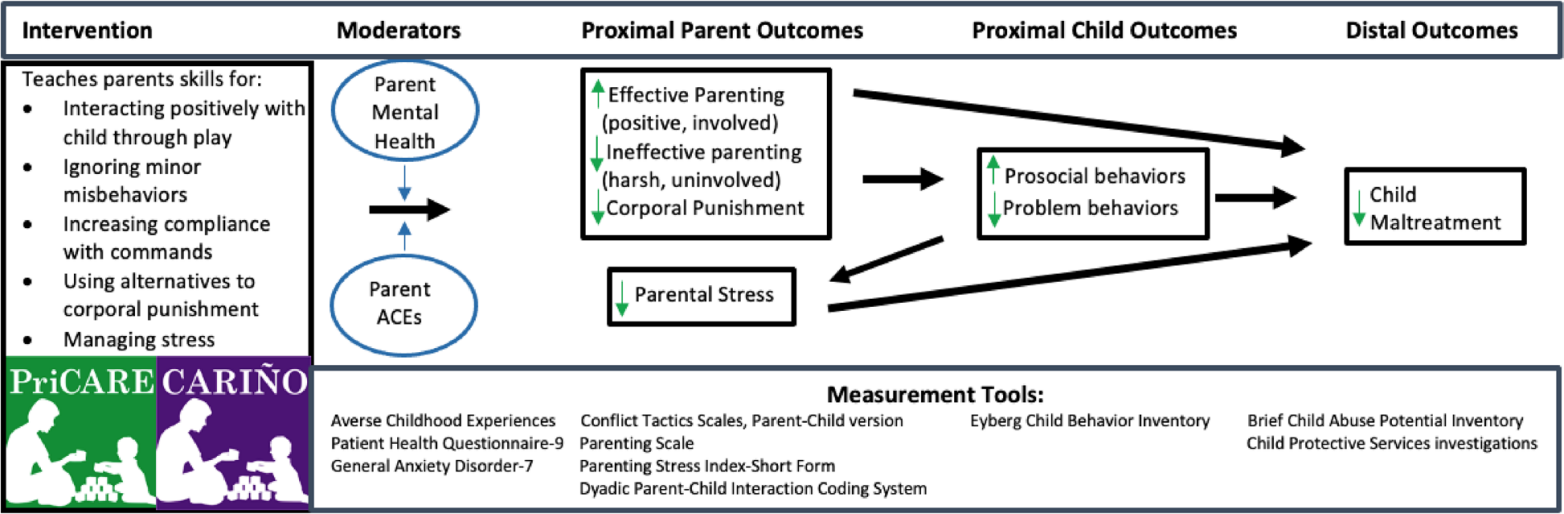
Conceptual model for PriCARE/*CARIÑO* intervention to prevent child maltreatment

Child-Adult Relationship Enhancement in Primary Care (PriCARE)/*Criando Niños con CARIÑO* is a pediatric primary care-based 6-session group parent training program for caregivers of children ages 2–6 years old. This manualized skill-based program, available in both virtual and in-person formats, aims to replace harsh, inconsistent, and permissive parenting with effective positive behavior management techniques. The use of positive behavior management techniques results in decreases in externalizing child behavior problems and stress related to parenting, which reduces the risk of neglect and of discipline escalating to corporal punishment and physical abuse (Fig. 1). All program components are standardized [36, 37] and supported by the underlying theory of change [29, 30] existing literature on evidence-based parenting interventions [27], and expert consensus [28].

Three randomized controlled trials demonstrate that PriCARE/*CARIÑO* decreases child behavior problems, harsh and permissive parenting, and parent stress, but we have not previously examined the occurrence of CM or CM risk scores as outcomes [38–41]. Furthermore, though we have addressed multiple implementation challenges [42], we have yet to rigorously assess implementation determinants (barriers/facilitators) related to referrals, enrollment, and attendance that can inform the development of strategies for implementing and scaling-up PriCARE/*CARIÑO.*

### Objectives {7}

*The primary objective* is to evaluate the relative effectiveness of PriCARE/*CARIÑO* in reducing the frequency of CPS investigations of suspected CM in the target population of low-income (has Medicaid/CHIP/no insurance), racially diverse, English- and Spanish-speaking families. To better understand the treatment effect, we will also explore potential moderators (measures of caregiver depression, anxiety, and adverse childhood experiences, {ACEs}) of the treatment effects.

*The secondary objective* is to evaluate the relative effectiveness of PriCARE/*CARIÑO* in the target population in terms of the following survey instruments:

- Reducing CM risk (BCAP)
- Reducing harsh/neglectful parenting (CTS-PC and PS)
- Reducing caregiver stress (PSI-SF)
- Improving child behavior problems (ECBI)
- Strengthening parent-child interactions (DPICS)

To better understand the treatment effects, we will also explore potential moderators (measures of caregiver depression, anxiety, and ACEs) of the treatment effects.

*The third objective* is to investigate mediation effects of harsh/neglectful parenting (CTS-PC and PS), parent stress (PSI-SF), and child behavior (ECBI) on the relationship between PriCARE/*CARIÑO* exposure and subsequent CPS investigations and CPS risk scores (BCAP). The analyses will be used to generate and refine hypotheses about mediation effects.

*The fourth objective* is to understand barriers and facilitators to PriCARE/*CARIÑO* implementation in pediatric primary care, specifically related to clinician *referral* of eligible dyads, caregiver *enrollment*, and caregiver *attendance* to develop targeted implementation strategies to test in a future trial.

*The fifth objective* is to conduct an ancillary observational study comparing the virtual delivery of the intervention to (historical) in-person delivery of the intervention. The ancillary analyses will be performed to guide a mid-study decision about whether to (A) continue virtual delivery of PriCARE/*CARIÑO* or (B) return to in-person delivery. In pre-pandemic studies, the intervention was delivered in-person to groups of 4–10 parents at the primary care clinic. During the COVID-19 pandemic, PriCARE/*CARIÑO* was adapted for virtual delivery; trivial changes to the program curriculum were made to accommodate the virtual platform, but no core components were altered. The ancillary study will investigate whether virtual delivery is as effective as in-person delivery. At the midpoint of the study, a decision (A vs. B) will be made based on the ancillary study results, as well as the acceptability and feasibility of the in-person and virtual group interventions.

### Trial design {8}

This is a randomized, controlled, investigator-blinded, multi-center, superiority trial of the PriCARE/*CARIÑO* intervention with two parallel groups and a primary endpoint of a CPS investigation during the time period 4 to 52 months after enrollment. In addition to investigating the effectiveness of the intervention, we will also explore barriers and facilitators to its implementation in pediatric primary care. The study is therefore a hybrid type-1 effectiveness-implementation trial [43]. The target enrollment is 1932 English- and Spanish-speaking caregiver-child dyads. Eligible children will be 2–6 years of age with Medicaid/CHIP/no insurance recruited from 10 to 15 pediatric primary care clinics. In each clinic, each dyad will be assigned by 1:1 randomization to one of the two regimens: PriCARE/*CARIÑO* plus usual care, or usual care alone.

## Methods: participants, interventions, and outcomes

### Study setting {9}

We will recruit from pediatric primary care clinics affiliated with three sites in the USA: University of North Carolina Health, WakeMed, and Children’s Hospital of Philadelphia. The clinics are in urban and suburban locations. Some clinics are associated with residency programs and include resident physicians and others are community practices without trainees. We anticipate recruiting from 10–15 clinics altogether although the exact number will depend on meeting enrollment goals.

### Eligibility criteria {10}

#### Child eligibility

The following child inclusion criteria will be used: (1) age 2–6 years old; (2) patient at participating pediatric primary care clinic; (3) has Medicaid/CHIP/no insurance; (4) lives in North Carolina or Philadelphia; (5) their caregiver/s have not previously participated in PriCARE/*CARIÑO*; (6) other 2–6 years old members of their immediate family have not been enrolled in PriCARE/*CARIÑO*.

Children meeting the following criteria will be excluded: (1) cognitive functioning below 2-year-old level, as measured by the screening questions; (2) diagnosis of or under evaluation for autism; (3) diagnosis of or under evaluation for oppositional defiant disorder; (4) receipt of individual behavioral health treatment or medication for a behavioral health problem; or (5) have caused physical injuries to themselves or another person on purpose more than once in the past 3 months. Children meeting these criteria will be excluded as they may benefit from a different intervention targeted to their specific needs (criteria 1 and 2), may require a more intensive individual treatment program (criteria 3 or 5), or are already receiving treatment (criterion 4). Previous CPS investigation of suspected CM is *not* an exclusion criterion.

#### Caregiver Eligibility

The following caregiver inclusion criteria will be used: (1) age 18 years and older; (2) fluency in English or Spanish; (3) is the legal guardian of child subject; (4) is available to participate when PriCARE/*CARIÑO* groups are offered; (5) has a cellular phone with text messaging capacity; (6) has demonstrated the ability to access the virtual platform during the consent process; (7) resides in the same household as the child for at least 50% of the time for the next 4 months; (8) has not previously participated in PriCARE/*CARIÑO*.

For objectives 3 and 4, we will interview a subsample of the study participants to investigate implementation determinants and to uncover possible mechanisms of change of the PriCARE/*CARIÑO* intervention. To be eligible for these interviews, participants must meet one of the following criteria: (1) caregiver enrolled in the study who was randomized to the intervention; (2) clinician at a study primary care clinic; or (3) facilitator administering the intervention.

### Who will take informed consent? {26a}

Trained research coordinators will contact the referred caregivers via phone and introduce the trial via script. If willing, the caregiver and child will be screened for eligibility and the study will be fully explained. If the caregiver is willing and eligible to participate in the trial, research coordinators will obtain written informed consent from the caregiver. For objectives 3 and 4, research coordinators will obtain informed consent from the clinicians and facilitators.

### Additional consent provisions for collection and use of participant data and biological specimens {26b}

We will inform caregivers during the consent process that we will keep some data for future research. Data will be stored in a password-protected database and any future studies using this data will be submitted to the IRB for review and approval. This trial does not involve collecting biological specimens.

## Interventions

There are two treatment regimens: usual care with and without the PriCARE/*CARIÑO* intervention.

All enrolled caregivers, regardless of the regimen assigned, will complete a common set of evaluations comprising survey data.

### Explanation for the choice of comparators {6b}

Usual care alone is the control regimen. Inclusion of the control regimen is required for evaluation of the relative effectiveness of PriCARE/*CARIÑO* in the setting of usual care. The main purpose of a control regimen is to control for threats to internal validity [44], and based on our study question, attention is not a threat to internal validity [45, 46]. An attention control might allow us to identify the components or combination of components (contact time, group interaction, content, skill mastery) that contribute to the reduction in the outcome. However, that is not the purpose of our study. Rather, our goal is to evaluate the effectiveness of PriCARE/*CARIÑO*. Many behavioral intervention trials do not control for attention [47, 48].

### Intervention description {11a}

Child Adult Relationship Enhancement in Primary Care (PriCARE)/*Criando Niños con CARIÑO* is a 6-session, manualized, skill-based, group parent training program offered to caregivers of toddlers and preschool-aged children in the pediatric primary care setting [36, 37]. PriCARE/*CARIÑO* groups are administered at different times/days to accommodate caregiver schedules. Each group is facilitated by 1 or 2 mental health professionals trained in the model (facilitators) and is attended by approximately 4–10 caregivers without their children. For this study, the intervention is delivered on a virtual platform and intervention handouts and a toy for home practice are mailed to the participant. Program materials are also emailed and texted to participants.

The PriCARE skills are taught in 6 weekly 80-min sessions. Sessions 1–4 teach parenting skills focused on giving attention to children’s positive, pro-social behaviors, while ignoring minor misbehaviors. Mastery of the 3 P skills (Praise, Paraphrase, and Point-out-Behavior) helps caregivers learn how to promote positive behaviors in their children. Sessions 5–6 teach techniques for giving children effective commands to set age-appropriate limits. The importance of play in supporting a child’s development and establishing a strong foundation for the relationship between the child and caregiver is emphasized. Caregivers are encouraged to practice the PriCARE/*CARIÑO* skills at home during brief (3–5 min) daily 1-on-1 play sessions with their child. PriCARE/*CARIÑO* includes a trauma and stress education component that contextualizes the use of these skills with the types of behaviors and problems exhibited by many children living with psychosocial adversity and chronic familial stress.

In addition to the 6 sessions, 3 text messages are sent following each session. The “Fact” message provides a piece of key content from that week’s session. The “Tip” message serves to reinforce the practice of a skill from that week’s home practice while also including a video demonstration of that skill. The “Encouragement/Reminder” message functions to automate attendance confirmations for the subsequent session as well as provide support and reassurance for the caregivers’ efforts in practicing that week’s skill.

The enrolled dyads will receive usual care with or without the PriCARE/*CARIÑO* intervention. For all dyads, *usual care* includes brief anticipatory guidance regarding behavior management and discipline during well child encounters. In some cases, when indicated due to problematic parenting or child behavior problems, providers may also choose to refer children for individual treatment. Some of the clinic sites in this study have social workers who provide case management, but do not directly provide therapeutic interventions to young children.

### Criteria for discontinuing or modifying allocated interventions {11b}

**Fig. 2:**
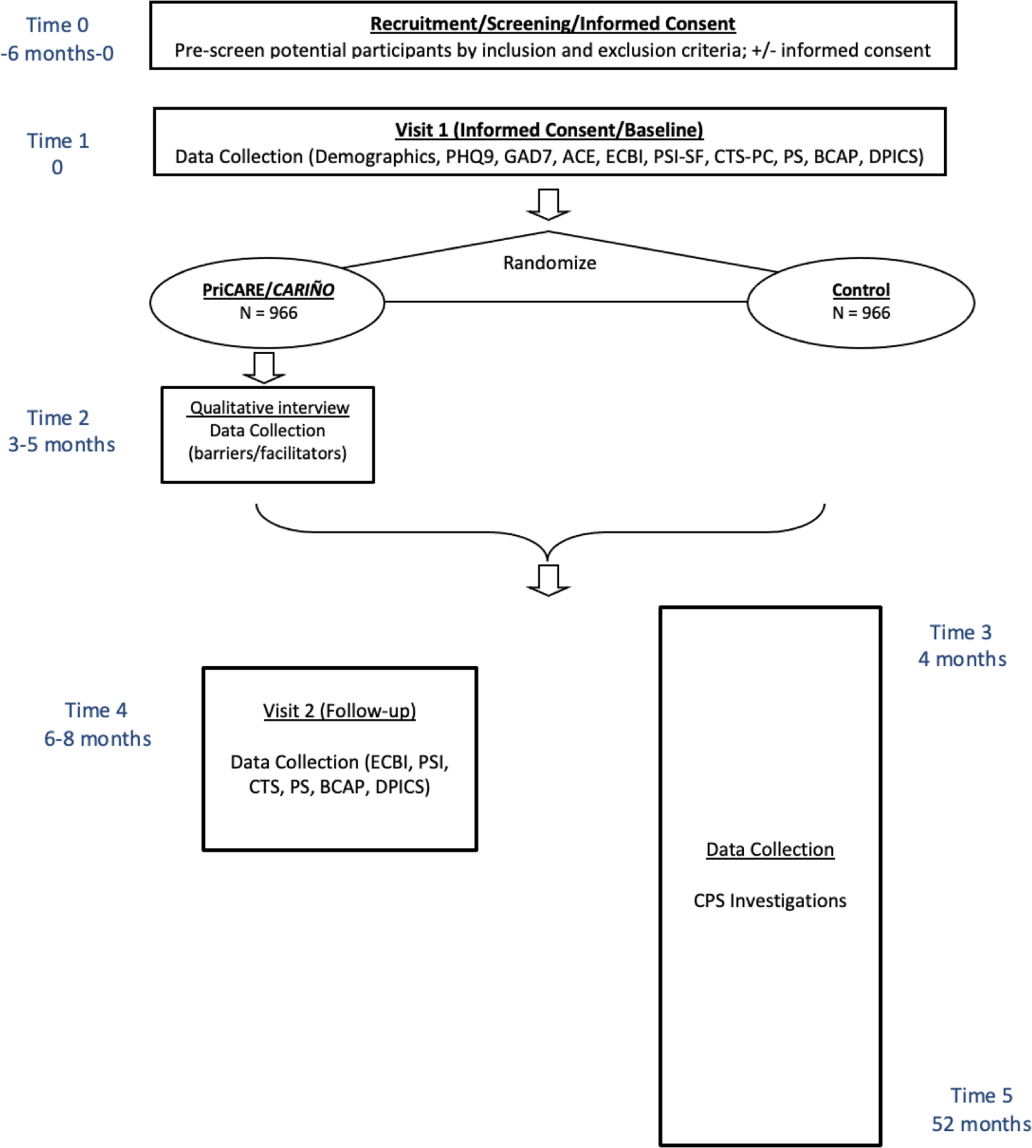
Flow of participants

If a caregiver misses 2 consecutive PriCARE/*CARIÑO* sessions, they will be removed from their current intervention group and invited to participate in a future intervention group if they are able to complete the group within the allowable timeframe (Fig. 2). If they do not attend a future group, data collection will proceed as planned without completion of the intervention. Even when a participant randomized to the intervention does not receive the intervention, we will attempt to complete the follow-up interview to ensure a complete dataset. Modification of the intervention may occur after an ancillary study is conducted mid-study (objective 5).

### Strategies to improve adherence to interventions {11c}

The trial will implement several strategies to improve adherence to the intervention, focusing on aspects related to both the facilitators and the caregivers.

#### Facilitator adherence to the PriCARE protocol

Fidelity promotion includes (1) standardized intervention materials and procedures [36, 37], (2) high-quality training of clinicians on the standardized protocol, (3) ongoing clinical consultation and feedback by senior PriCARE facilitators, and (4) ongoing fidelity monitoring.

##### PriCARE facilitator training

PriCARE facilitators are masters- or doctoral-level mental health professionals who complete a structured 16-h PriCARE training led by 2 senior PriCARE facilitators. The PriCARE group intervention has a standardized delivery format detailed in the PriCARE/*CARIÑO* manual [36, 37]. The PriCARE manual, video demonstrations, and text message algorithm are reviewed in detail at the PriCARE facilitator training. Following the structured training, the newly trained PriCARE facilitators continue to meet individually or in small groups with a senior PriCARE facilitator to review key program skills, concepts, and goals. The accreditation process is completed when the facilitator demonstrates the skill-based competency standards described below.

##### PriCARE accreditation

During the 16-h structured training and follow-up meetings, each trainee will be evaluated by a senior PriCARE facilitator to ensure mastery of (1) the use of the PriCARE skills; (2) coaching of PriCARE skills; and (3) presenting the material. PriCARE facilitators may begin co-facilitating groups with ongoing consultation following PriCARE accreditation.

##### PriCARE facilitator maintenance meetings

All PriCARE facilitators, regardless of experience, attend a yearly PriCARE review session in which common fidelity deviations are addressed and process/protocol clarifications are reviewed.

##### PriCARE consultation

Once a PriCARE facilitator starts co-facilitating PriCARE groups, they participate in weekly 1-h consultations with a senior PriCARE facilitator following each session. During consultations, session delivery, model fidelity, and progress toward mastery of PriCARE facilitation skills are reviewed in detail. Consultation is held with PriCARE facilitators for at least the initial 3 groups they co-facilitate, and until mastery is achieved as determined by fidelity monitoring and assessment of a senior PriCARE facilitator.

##### PriCARE fidelity monitoring

One randomly selected session from each group is observed and coded by a trained coder. Coding includes two facilitator categories (content and delivery), and one caregiver category (degree of caregiver participation). Fidelity codes and monitoring procedures are detailed in the PriCARE/*CARIÑO* manual [36, 37].

#### Caregiver adherence to the PriCARE protocol

Caregiver adherence will be promoted as follows: (1) caregivers who indicated that they are not willing and able to adhere to the study protocol will not have been enrolled; (2) text message/email reminders will be sent the day before and the day of each session with a session link; (3) caregivers who miss a session will be texted and emailed a link to a make-up video of the session and asked to watch it prior to the next session; (4) caregivers who miss 2 consecutive sessions will be invited to participate in a future intervention group if they are able to complete that group within the allowable timeframe (Fig. 2); (5) caregivers will be encouraged to call or text a study team phone with questions.

### Relevant concomitant care permitted or prohibited during the trial {11d}

No concomitant care or interventions are prohibited during the trial.

### Provisions for post-trial care {30}

We do not anticipate that any healthcare needs will arise as the result of this trial and do not anticipate a need for the provision of ancillary care due to trial participation.

### Outcomes {12}

#### Covariates

Following enrollment, the *demographic variables* to be recorded for the caregiver will include gender, date of birth, relationship to child subject, race, ethnicity, preferred language (Spanish or English), education level, annual household income, whether they identify as a single parent, and the number of children in their care. *Demographic variables* to be recorded for the child include gender, date of birth, race, and ethnicity.

Following enrollment, potential intervention *moderators* will be measured for each caregiver including caregiver depression/anxiety [49, 50] and caregiver history of childhood trauma [51, 52]. *Patient Health Questionnaire-9 (PHQ9)* is a 9-item self-report measure of depression symptoms and *Generalized Anxiety Disorder-7 (GAD7)* is a 7-item self-report measure of anxiety symptoms. Both instruments measure symptoms experienced during the past 2 weeks and both have excellent psychometric properties [53, 54]. *Adverse Childhood Experiences (ACEs) Questionnaire* is a 10-question self-report survey that assesses childhood exposure to abuse (physical, psychological, sexual), neglect (physical, emotional), and household dysfunction (mental illness, substance abuse, domestic violence, incarceration, and parental loss) [4].

#### Objective 1 outcome measures

Beginning 4 months after randomization, each caregiver-child dyad will be followed for a period of 12–48 months to document all CPS investigations of suspected CM. The length of the follow-up interval will depend on the dyad’s time of enrollment in the study. The primary outcome is a binary indicator of whether the caregiver-child dyad experienced at least one CPS investigation during follow-up. Investigations (unsubstantiated and substantiated) of CM rather than substantiations will be used because children with unsubstantiated reports and those with substantiated reports face similar rates of negative outcomes such as CM re-reports, negative impact on school achievement, and mortality [55–59].

#### Objective 2 outcome measures

CM risk, harsh, neglectful, and dysfunctional parenting, child behavior problems, caregiver stress, and caregiver-child interactions (*n*=120) will be measured prior to randomization and 6-8 months later. The anticipated result is that the intervention will improve these scores to varying degrees. See Table 1 and Fig. 2 for data collection timeline.

**Table 1:** Schedule of evaluations for quantitative objectives 1–3

Variables measured	Obj 1	Obj 2	Obj 3	Instrument	Occasions (months)		
					0	6–8	4–52
Demographics	Covariates	Covariates	Covariates	Self-report for caregiver and child	✓		
Spanish-speaking	Covariate	Covariate	Covariate	Self-report for caregiver	✓		
Caregiver depression	Moderator	Moderator		Patient Health Questionnaire-9 (PHQ-9)	✓		
Caregiver anxiety	Moderator	Moderator		General Anxiety Disorder-7 (GAD-7)	✓		
Caregiver ACEs	Moderator	Moderator		Adverse Child Experiences (ACE)	✓		
Child behavior problems		Dependent	Mediator	Eyberg Child Behavior Inventory (ECBI)	✓	✓	
Harsh parenting		Dependent	Mediator	Conflict Tactics Scales, Parent-Child version (CTS-PC)	✓	✓	
Dysfunctional parenting		Dependent	Mediator	Parenting Scale (PS)	✓	✓	
Caregiver-child interactions^b^		Dependent		Dyadic Parent-Child Interaction Coding System (DPICS)	✓	✓	
Caregiver stress		Dependent	Mediator	Parenting Stress Index-Short Form (PSI-SF)	✓	✓	
Maltreatment risk		Dependent	Dependent	Brief Child Abuse Potential Inventory (BCAP)	✓	✓	
Child maltreatment^**a**^	Dependent		Dependent	Child Protective Services (CPS) investigation			✓

^a^The length of follow-up data collection for CPS investigations will vary by participant depending on time of enrollment but will be up to 48 months (52 months after enrollment)

^b^Recorded for a subsample of 120 dyads

*CM risk will be measured by the Brief Child Abuse Potential Inventory (BCAP)*, a questionnaire that measures CM risk and includes a 34-item Child Physical Abuse scale [60]. The two internal validity scales can be used to create validity subtests: lie and random response [60]. BCAP is a brief version of the Child Abuse Potential Inventory and on cross-validation, BCAP risk scale scores demonstrated an internal consistency of 0.89 and substantial correlation with the Child Abuse Potential Inventory abuse risk score (r=0.96) [60, 61].

*Harsh and neglectful parenting will be measured by the Conflict Tactics Scales, Parent-Child version (CTS-PC)*, a 35-item scale focusing on the respondent’s behavior with their child including discipline methods. In this study, we will include the 18 items that constitute the nonviolent discipline, psychological aggression, and corporal punishment subscales [62–64]. We will not include the neglect, sexual abuse, physical assault, or physical maltreatment subscales of the CTS in order to minimize the potential legal risk to caregivers related to mandatory reporting laws. Reliability coefficients are moderate, with alpha coefficients of 0.70 for nonviolent discipline, 0.68 for psychological aggression, and 0.72 for corporal punishment [62–64]. The CTSPC has adequate discriminant and construct validity [62, 63]. Test-retest reliability has been established with a correlation of 0.8 at 5 months [62].

*Dysfunctional discipline will be measured by the Parenting Scale (PS)*, a 30-item self-report questionnaire designed to assess dysfunctional parenting discipline strategies including laxness (permissive inconsistent discipline, providing positive consequences for misbehavior), over-reactivity (harsh, emotional, authoritarian discipline characterized by irritability), and hostility (use of verbal or physical force). The PS has demonstrated adequate psychometric properties including internal consistency, test-retest reliability, and convergent validity with other validated measures, and is correlated with observational measures of inadequate parental discipline and child misbehavior [65–67].

*Child behavior problems will be measured by the Eyberg Child Behavior Inventory (ECBI)*, a 36-item parent survey used to assess the current frequency and severity of disruptive behaviors at home in children 2–16 years [68]. The test-retest reliability of the ECBI has been established with correlations near 0.75 for both subscales over a 10-month interval [69]. ECBI scores are highly correlated with observational measures of child negative affect, non-acceptance, and dominance [70]. The ECBI is also correlated with other measures of child behavior problems (has convergent validity) and conversely the ECBI has high discriminant validity. The ECBI is a sensitive indicator of intervention efficacy/effectiveness [71, 72].

*Caregiver stress will be measured by the Parenting Stress Index-Short Form (PSI-SF)*, a 36-item symptom inventory that can be used to identify caregiver-child dyads who are experiencing stress and at risk for dysfunctional parenting and behavior problems [73]. Psychometric evaluation of the PSI has demonstrated excellent internal consistency with a Cronbach’s alpha of 0.91 and excellent test-retest reliability with a Cronbach’s alpha of 0.84 for total stress [74].

*Caregiver-child interactions will be measured by the Dyadic Parent-Child Interaction Coding System (DPICS),* a coding system for specific structured caregiver-child interactions targeted by PriCARE [75]. DPICS includes codes for parent (commands, criticism, labeled praise) and child (noncompliance, compliance) behaviors. Inter-rater reliability, test-retest reliability, and convergent and discriminant validity are high [76, 77]. Caregivers and children are observed during two 5-min standard situations with instructions that produce opportunities to demonstrate differing levels of parental control: child-led play and clean-up. For a subsample of 120 enrolled dyads, these interactions will be video-recorded and coded by a team member trained to code DPICS and blinded to the study regimen.

#### Objective 3 outcome measures

We will investigate the roles of harsh/neglectful/dysfunctional parenting (CTS-PC and PS), parent stress (PSI-SF), and child behavior (ECBI) as potential mediators of the relationship between exposure to the PriCARE/*CARIÑO* intervention and outcomes of interest: occurrence of CPS investigations and CM risk scores (BCAP). The survey scales will be measured prior to randomization and 6-8 months later.

#### Objective 4 outcome measures

In addition to effectiveness outcomes, we will explore the relationship between implementation determinants (i.e., barriers and facilitators) and key implementation outcomes, including referral rates, enrollment rates, and attendance.

##### Implementation determinants

Semi-structured interviews, guided by the Theoretical Domains Framework (TDF) will be used to understand implementation determinants of PriCARE/*CARIÑO* across varied clinical settings [78–80]. The TDF is an ideal framework for our study because, in addition to being applied to explore barriers and facilitators to clinician implementation of evidence-based behaviors [81], it has also been extended to understanding patient behaviors [82], to identify mechanisms of change [83–85], and to identify and design strategies to address implementation barriers [86].

##### Referral rates

*Referral rates* will be measured at the clinic level and will be defined as the number of patients referred to PriCARE/*CARIÑO* divided by the number of eligible patient encounters during the study period. Referrals will be auto-captured through the Electronic Medical Record (EMR) and will be divided by the total number of eligible patient encounters. Eligible patient encounters will be defined as well visits for children 2 to 6 years old who have Medicaid, CHIP, or no insurance and whose caregivers speak English or Spanish and have not previously participated in PriCARE/*CARIÑO*.

##### Enrollment rates

*Enrollment rates* be measured at the clinic level and will be defined as the number of dyads who are referred and enroll in the study divided by the total number of eligible dyads referred to the study.

##### Attendance

*Attendance* will be recorded by research coordinators and will include the number of sessions (0–6) attended by each caregiver randomized to PriCARE/*CARIÑO*. Mean attendance will be calculated for each clinic.

#### Objective 5 outcome measures

This observational ancillary study will focus on longitudinal changes in the Eyberg Child Behavior Inventory (ECBI) Intensity Scale score (range 36–252). Summed across 36 items, lower scores are more favorable. The ECBI was the primary outcome measure in the three prior randomized clinical trials (historical data) and is a secondary outcome in this study (prospectively observed).

### Participant timeline {13}

The participant timeline is shown in Fig. 2.

### Sample size {14}

The study to be conducted in 10–15 clinics will enroll 1932 eligible caregiver-child dyads drawn from the target population of low-income children 2–6 years of age. We anticipate that about 15% of the caregivers will be Spanish speakers. We anticipate that about 200–300 of the 1932 enrolled dyads will experience CPS investigation during the study after 1:1 randomization stratified by clinical site and language. In designing the study and choosing the target sample size, considerations included (1) information from previous studies; (2) the availability of eligible dyads, clinics, and clinic personnel; (3) costs and time requirements; (4) anticipated frequencies of drop-out, missing data values, and nonadherence to protocol; (5) the statistical analysis strategies and methods specified for each of the five objectives; (6) anticipated levels of precision of estimators of key population parameters (estimands); and (7) anticipated levels of power of a hypothesis test procedure concerning the effectiveness of the intervention.

#### Objective 1 considerations

Considerations for Objective 1 were informed by the following published reports: during a 43-month study of 250 children in Baltimore (92% Medicaid), 19% had at least one CPS investigation [87]. In a state-wide prospective observational study in California, 21% of children with Medicaid at birth had at least one CPS investigation over 5 years [58]. In Cleveland, from birth to age 10, 31% of children were investigated by CPS [88]. An estimated 37% of all US children will have been investigated by CPS by the time they reach age 18 years [1]. These reports suggested that if our target population receives usual clinical care only and each child is followed for 12 to 48 months thereafter, then about 20% of the children would experience at least one CPS investigation during follow-up.

Assumptions: The statistical analyses will rely on generalized logistic regression models with *clinical site* treated as a categorical random effect, and with fixed effects representing *length of follow-up* (log_10_ months), *treatment regimen*, and *language* (Spanish-speaking). Additionally, variations on the model will be used to explore additional fixed effects; e.g., demographic characteristics, caregiver scores (*PHQ9, GAD7, ACEs*) at enrollment, and terms representing interactions among the fixed-effects.

To avoid model overfitting bias, we require at least 20 cases (20 dyads experiencing CPS investigation) per fixed-effect regression coefficient included in the model; e.g., if 200 cases occur, the models can support up to 10 coefficients in the regression equation. We anticipate that the chosen sample size is sufficient and appropriate for supporting objective 1.

For considerations of precision and power, simplifying assumptions and conjectures were made:

- the proportion (π_1_) experiencing CPS investigation is 20% in the target population when receiving only usual care, the proportion (π_2_) is 13% when the target population receives usual care plus the PriCARE/*CARIÑO* intervention, the follow-up interval is the same for all the small groups, and that intraclass correlation due to dyad clustering of dyads is small (*ρ* ≤ 0.15). In regard to precision, discussed in terms of the widths of 95% confidence intervals (CI): if the observed proportion of cases for the control regimen happens to be 20%, then the observed 95% CI for π_1_ would be {20.0% ± 4.0%}; if the observed proportion for the intervention regimen is 13%, then the observed 95% CI for π_2_ would be {13.0% ± 3.3%}; and the observed 95% CI for the difference (π_1_ − π_2_) would be {7.0% ± 4.5%}, approximately. In regard to power for the test of H_o_ “the treatment effect (π_1_ − π_2_) is exactly zero in the target population,” if (π_1_ − π_2_) = 7.0% then there is an 80% chance that a sample of dyads will be drawn from the target population such that a small *p*-value (< *α*=0.05) will be observed. If H_o_ is true then the power level is 5%.

#### Objective 2 considerations

We conjecture that the intervention improves proximal outcome scores: CM risk, child behavior problems, caregiver-child interactions (*n*=120), caregiver stress, harsh parenting by the caregiver, and dysfunctional parenting by the caregiver all measured prior to randomization and 6–8 months later. Based on prior studies we anticipate a 10% loss to follow-up at the 6-month interview.

#### Objective 3 considerations

The investigation of mediation effects will focus on model-building, estimation of effects, and qualitative analysis for purposes of hypothesis generation/refinement. We anticipate that the study design and target sample size will be adequate for these exploratory analyses.

#### Objective 4 considerations

A sample of 6 clinics will be selected, including 3 high- and 3 low-implementing clinics as identified by their clinic referral rate, enrollment rate, and mean attendance. Samples of participants will be drawn from each of the 6 clinics. To ensure that the data reflect the diversity of roles and experiences, we will use purposive sampling to include caregivers, clinicians, and PriCARE/*CARIÑO* facilitators. Caregiver sampling will be balanced by language and number of sessions attended (<3 or >3). Clinician sampling will be balanced to include those with high and low referral rates. Altogether, we anticipate interviewing 3–5 caregivers per site (18–30 total), 2–3 clinicians per site (12–18 total), and 10 PriCARE/*CARIÑO* facilitators. We anticipate this will provide sufficient data to achieve saturation relative to our primary objectives of identifying factors that may be implementation determinants (objective 4) and contextualizing the quantitative analyses of mechanisms of change (objective 3) [89].

#### Objective 5 considerations

Pre- and post-intervention ECBI scores for 483 enrolled dyads (randomized 1:1) studied during a time of virtual delivery of the PriCARE/*CARIÑO* intervention will be compared to historical scores from 444 dyads (randomized 1:1) previously studied in three trials during in-person delivery. The observational cohort-comparison study may be subject to confounding biases. The analyses will be descriptive with a focus on point and interval estimates of means, mean changes, differences, and variance components. In terms of the ECBI pre- vs post-intervention change score, a 10-unit difference between the two regimens will be considered clinically important.

### Recruitment {15}

**Fig. 3:**
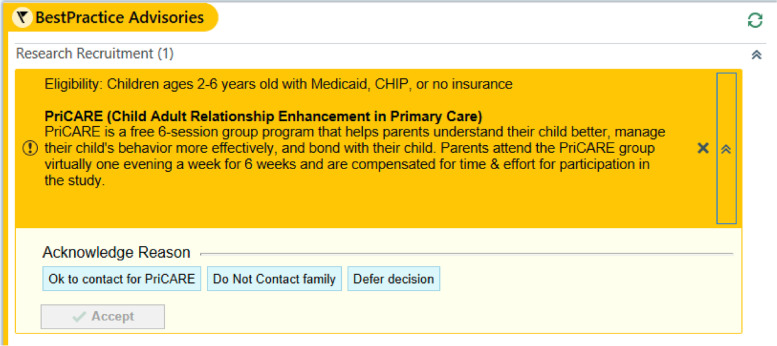
PriCARE electronic medical record alert that reminds clinicians to refer eligible patients during well child visits

The study team will be made aware of potential participants in 3 ways: (1) A point-of-care alert in the EMR will prompt physicians to inform caregivers at eligible well child visits about the PriCARE/*CARIÑO* study and if the caregiver provides verbal permission to be contacted by study staff, a referral message will be sent to the study team (Fig. 3); (2) Clinic providers will obtain verbal permission from the caregiver to be contacted by study staff and notify the study team of the caregiver contact information in-person, via EMR message, via EMR order, email, or phone; (3) The interested caregiver may contact the study team directly by email or phone using the study team’s contact information provided on the recruitment flyer, which will be posted in the waiting room and in exam rooms and included in well child visit packets.

## Assignment of interventions: allocation

### Sequence generation {16a}

Enrolled dyads will be assigned by 1:1 stratified randomization to PriCARE/*CARIÑO* plus usual care or to usual care alone. The randomization schedule, stratified by *clinical site* and *language*, will be generated using permuted blocks of sizes 2 and 4 in an unpredictable order. This means that prior to the start of recruitment we will have created two separate allocation tables for each clinical site. Each allocation table will be sufficiently extensive to accommodate an unexpectedly large cohort. The allocation tables will be concealed from the study personnel during the study.

### Concealment mechanism {16b}

The collection of site-and-language-specific allocation tables will be uploaded to the REDCap database prior to study initiation. A feature of the REDCap database will be used to conceal the treatment assignments from the study team until the moment the newly enrolled caregiver-child dyad is ready to be assigned to a regimen.

### Implementation {16c}

During recruitment, a Study ID number will be assigned to each dyad. Screening for eligibility will be conducted by phone after verbal informed consent to be screened is obtained. Eligible dyads who wish to enroll will provide written informed consent to study participation and complete the first REDCap data collection survey. The REDCap randomization tool will be used to assign the enrolled dyad to a treatment regimen: a study team member will click on the “randomize” field to reveal the allocation and notify the caregiver of their treatment regimen.

## Assignment of interventions: blinding

### Who will be blinded {17a}

Due to the nature of the intervention, subjects cannot be blinded to regimen allocation but will be instructed not to disclose their allocation status at the follow-up assessments. Regarding the primary outcome, CPS investigations of suspected CM, CPS agencies, and administrators recording reports and preparing data files with CPS data for the study team will be blind to treatment allocation. With regard to the secondary outcomes, baseline interviews will occur prior to randomization and all follow-up data collection will be conducted by an assessor blind to treatment allocation. For each member of the study team, REDCap data entry and access privileges will be personalized to the role of the individual such that team members will only be able to view/access specific fields within records in order to maintain blinding.

### Procedure for unblinding if needed {17b}

Given that subjects are already unblinded to treatment allocation, a procedure for further unblinding is not applicable as we anticipate no situation in which unblinding of study team members will be necessary.

## Data collection and management

### Plans for assessment and collection of outcomes {18a}

The Research Data Capture (REDCap) software system will be used to create and manage the study databases. Multiple streams and types of data will be collected including CPS investigations, surveys with a REDCap user interface, caregiver-child observations, qualitative interviews, attendance data, and data regarding intervention referrals, eligibility, enrollment, and fidelity.

#### CPS investigations

Through Data Use Agreements with the relevant CPS agencies, the research team will provide a matching dataset to CPS containing caregiver and child demographic information required for matching and the start and end date for CPS data matching for each dyad. Using this information, a coded dataset, purged of child and caregiver identifiers, containing study ID and data on investigated reports (report date, allegation type, investigation outcome) occurring during the CPS data matching period will be created for the study. The CPS dataset will be merged with a coded dataset containing demographic and survey data from REDCap but purged of PHI. The two datasets will be merged on the study ID number, and then fully de-identified for final analysis.

#### REDCap enabled surveys

Trained research coordinators will collect survey data via phone or videoconference at baseline (0 months) and at follow-up (6–8 months). The caregiver may also choose to complete the survey online. The surveys will include information on demographic characteristics, potential moderators (caregiver mental illness and caregiver history of childhood trauma), and measurements of outcomes of interest detailed in Table 1. Participants will be compensated $40 for each REDCap survey.

#### Caregiver-child observations

In addition to the caregiver-reported survey data, 120 dyads will complete the DPICS at baseline and at follow-up (6–8 months). Starting in year 3 of the study, we will consecutively administer the DPICS until 120 dyads have completed both the baseline and follow-up DPICs. Participants will be compensated $20 and a toy for each DPICS.

#### Qualitative Interviews

Qualitative interviews will be conducted with caregivers randomized to PriCARE/*CARIÑO*, referring clinicians, and PriCARE/*CARIÑO* facilitators from 3 low-implementing and 3 high-implementing clinics. TDF will inform interview guide development and transcript coding and analysis. TDF is an integrated theoretical framework with 14 domains that focus on the cognitive, affective, social, and environmental influences on behavior [78, 79]. The research team will select the most relevant domains to the behaviors of interest (referrals, enrollment, and attendance) in designing the interview guide. The goals of the interviews include: (1) examine implementation determinants and characterize strengths and barriers across settings; (2) solicit feedback regarding lessons learned and best practices to support wide-based dissemination and implementation; and (3) identify perceived mechanisms of change of the intervention itself. Participants will be compensated $20 for each interview.

#### Intervention referral, enrollment, and attendance

Clinician referrals to PriCARE/*CARIÑO* from the EMR alert and the number of patient encounters in which the EMR alert was active will be used to calculate clinic referral rates which will be stored in the REDCap database. Enrollment and attendance will be recorded by research coordinators and will be used to calculate clinic means. Data will include the number of referred dyads who enroll in the study and the number of sessions attended by each caregiver randomized to PriCARE/*CARIÑO*.

### Plans to promote participant retention and complete follow-up {18b}

The reasons for missing data values will be documented in the REDCap database. We will attempt to follow up all enrolled participants, even if they withdraw from their assigned regimen.

Caregiver retention in the study will be promoted as follows: (1) compensate dyads immediately following data collection; (2) obtain multiple phone numbers, email addresses, and mailing addresses from dyads upon enrollment to ensure the ability to contact for follow-up data collection; (3) call/email all contact information multiple times at different times of the day to complete follow-up data collection interview; (4) provide multiple time/date options and modalities (phone, virtual, online self-administered, in-person) to complete data collection; (5) provide direct study team phone number for study participants to call or text with study questions or to schedule study interviews.

### Data management {19}

Multiple features will ensure both the integrity and validity of the collected data. Referral, enrollment, attendance, fidelity, DPICS codes, and caregiver interview data will be entered and stored in electronic REDCap databases.

The caregiver survey data will be directly entered into the data collection form thereby eliminating risk of errors in the transposition of data. REDCap provides real-time validation rules (with automated data type and range checks), easy data manipulation (with audit trails for reporting, monitoring, and querying subject records), and an automated export mechanism to common statistical packages. Team members involved with data entry will undergo extensive training on the study database and best practices for data entry including mock data entry trainings.

Video recordings, audio recordings, and interview transcripts will be stored on a secure password-protected server. As qualitative data collection progresses, the study team will review interview transcripts to assess data quality and ensure the content collected meets our study aims.

De-identified data from CPS will be stored on a secure password-protected server. After all data collection is completed, the CPS data will be merged with the REDCap data on the study ID. The study ID will be replaced with a new randomly generated unique identifier that cannot be traced back to the REDCap data. The de-identified, coded dataset will be used for analysis.

### Confidentiality {27}

All data and confidential study files will be stored in (1) a secure, web-based data application (REDCap) that is password protected so that only study team members granted privileges will have access or (2) single sign-on secure access networks in study-specific folders accessible only to study team members. All personal identifying information will be removed before a de-identified finalized, merged dataset for analysis is provided to the statistical team.

### Plans for collection, laboratory evaluation, and storage of biological specimens for genetic or molecular analysis in this trial/future use {33}

Biological specimens will not be obtained in this trial.

## Statistical methods

### Statistical methods for primary and secondary outcomes {20a}

#### Analysis plans for objective 1

##### Evaluation of effectiveness

For the primary analysis of the proportion of children with at least one CPS investigation during follow-up, we will use a logistic regression model with covariate adjustment for the length of follow-up (log_10_ months). To account for the clustering of dyads within clinical sites and within small groups of 8-10 for PriCARE sessions, the model will be fitted using generalized estimating equation (GEE) methods with robust standard error estimates. *Clinical site* will be treated as a random effect. To improve precision of the estimators of *regimen*-specific treatment effects, caregiver *language* (Spanish speaking) will be included as a covariate. This model assumes that the difference between regimens is not a function of *language*. The population parameters (estimands [90]) of interest are the regression coefficients, the regimen-and-language-specific proportions that would be expected during 12–48 months of follow-up, and the treatment effect (expected difference between proportions) along with its odds ratio. The point estimates and 95% CI estimates for these estimands will be presented graphically in a figure using a forest-plot format. The p-value for the treatment effect will be shown to four decimal places and will be interpreted as a continuous score [91–93]. The *S*-value = log_2_ (^1^/_*p* value_) representing the available amount of information (Shannon information) against the null hypothesis “the treatment effect is exactly zero” will also be shown in the figure.

##### Effectiveness as a function of language

A *regimen*-by-*language* interaction term will be added to the above model to allow evaluation of intervention effectiveness as a function of *language*. A forest plot figure will display point and interval estimates for the estimands: proportions, differences of proportions, and the two language-specific treatment effects and their difference along with odds ratios. *P*-values and *S*-values for the two treatment effects and for their difference will be included in the figure to address (null) hypotheses such as “among Spanish-speakers the treatment effect is zero,” “both treatment effects are zero,” and “the two treatment effects are exactly the same (no effect-modification).”

##### Hypothesis generation/refinement

To enhance understanding of the main results (above) and to explore the roles of other covariates, we will explore numerous versions of the logistic model. These will include additional covariates measured prior to randomization such as the child (e.g., age, gender, race, ethnicity) and caregiver (e.g., age, gender, race, ethnicity, education, number of children) demographic variables, and caregiver scores (e.g., PHQ9, GAD7, ACEs). Moderating effects of these covariates will be investigated by including interaction terms such as PHQ9-by-regimen, GAD7-by-regimen, and ACEs-by-regimen. Overfitting bias due to inclusion of too many covariates will be avoided in these exploratory model-building efforts. We anticipate observing at least 200 “cases” (children with one or more CPS investigation) which is sufficient to support the fitting of this model with 10 fixed-effect coefficients representing independent variables. If 300 cases occur, then the logistic model would support up to 15 coefficients.

#### Analysis plans for objective 2

##### Evaluation of effectiveness

For each of the secondary outcome variables (BCAP, CTS-PC, ECBI, PSI-SF, PS, DPICS) the analyses will rely on a generalized linear model with GEE estimation. The dependent variable will be the change score computed as the follow-up score (at 6–8 months post-randomization) minus the baseline score. The independent variables will be *regimen* and *language* (Spanish-speaking). *Clinical site* will be treated as a categorical random effect. The estimands of interest are mean levels of the scores. The analysis strategies will be very similar to those described above in the “Analysis plans for objective 1” section. Point and interval estimates of the estimands will be presented graphically in forest plot figures.

##### Effectiveness as a function of language

The generalized linear models for mean change scores will be modified to include a *regimen*-by-*language* interaction term. The analysis strategies will be very similar to those described in the “Analysis plans for objective 1” section—albeit models for mean change scores rather than models for expected proportions.

##### Hypothesis generation/refinement

To enhance understanding of the main overall assessments of effectiveness and to explore the roles of other covariates, we will explore numerous versions of the models for mean change scores. The analysis strategies will be very similar to those described in the “Analysis plans for objective 1” section—albeit models for mean change scores rather than models for expected proportions.

#### Analysis plans for objective 3

We will investigate mediation effects of harsh/neglectful parenting (CTS-PC and PS), parent stress (PSI-SF), and child behavior (ECBI) (*mediators*) on the relationship between PriCARE/*CARIÑO* (*exposure*) and CPS investigations/CPS risk (BCAP) (*outcomes*). Generalized linear model methods similar to those specified for objectives 1 and 2 will be used to investigate direct associations between exposures mediators and mediators outcomes. The exposure-mediator-outcome triplets that demonstrate clinically important magnitudes of effect will be further investigated for mediation. Estimation of the average causal mediation effect, average direct effect, total effect, and proportion mediated and their corresponding 95% CIs will be computed via bootstrap using 500 resamples. In addition, triangulation [94] will be used to contextualize the results of this mediation analysis with the results of the qualitative analysis (described below in the “Analysis plans for objective 4” section) of the intervention’s possible mechanisms of change. These model-building investigations will result in hypothesis generation/refinement.

#### Analysis plans for objective 4

In the last year of the study, clinics will be characterized according to clinician referral, caregiver enrollment, and caregiver attendance rates and 3 high-implementing and 3 low-implementing clinics will be selected for in-depth study. Guided by TDF [78, 79], semi-structured interviews of clinicians, PriCARE/*CARIÑO* facilitators, and caregivers will be conducted at each of the selected clinics. Purposive sampling will be used to capture clinicians with high (top quartile) and low (bottom quartile) referrals and caregivers with high (>3 sessions) and low (≤ 3 sessions) attendance from each clinic. Altogether, we anticipate interviewing 3–5 caregivers per site (18–30 total), 2–3 clinicians per site (12–18 total), and 10 PriCARE/*CARIÑO* facilitators.

Interviews will continue until thematic saturation is reached. Transcribed interviews will be analyzed using qualitative content analysis, a theory-driven approach [95, 96]. This coding approach is both deductive (codes are derived from TDF) and inductive (codes are derived from the data). The goals of the analyses will be to identify barriers and facilitators associated with implementation, to identify targeted implementation strategies that can be evaluated in a future trial, and to identify potential mechanisms of intervention-induced change.

#### Analysis plans for objective 5

The ancillary study will be conducted when 483 enrolled caregiver-child dyads (randomized 1:1) have completed the 6-month follow-up interview. Pre- and post-intervention ECBI scores for 483 enrolled dyads (randomized 1:1) studied during a time of virtual delivery of the PriCARE/*CARIÑO* intervention will be compared to historical scores from 444 dyads (randomized 1:1 or 2:1) previously studied in 3 trials during in-person delivery. As in the case of objective 2, the analyses will rely on a generalized linear model with GEE estimation. The dependent variable will be the ECBI change score computed as the 6-month follow-up score minus the baseline score. Participants lost to follow-up will have missing values. The independent variables will be *treatment regimen* (intervention, control) and *clinical site*. Point estimates with 95% CI estimates will be obtained for the population parameters of interest: total variance, mean change for each regimen, the difference between the two mean changes. The treatment-effect estimates will be presented graphically in forest plot figures. Comparable results will be obtained using the dyad-level data from each of the 3 previous trials. To obtain point and interval estimates of differences among the four studies, the generalized linear model will be expanded to include data from all 4 studies along with use of terms in the regression equation representing *study* and *study*-by-*regimen* interaction. The means and mean differences of interest include those that address regimens, studies, differences between regimens (i.e., treatment effects), and differences between studies. The various study-specific estimates of means, mean differences, and variances will be presented graphically in forest plot figures.

#### Interim analyses {21b}

Mid-study inspection of the data will not be used to stop the study early and will not be used to change the enrollment sample size. The ancillary study of treatment effects in terms of ECBI change scores has the potential to modify the intervention regimen from virtual delivery to in-person delivery of PriCARE/*CARIÑO*.

### Methods for additional analyses (e.g., subgroup analyses) {21b}

#### Sensitivity analyses objective 1

To guide our level of trust in the main results about effectiveness, sensitivity analyses will be used to evaluate the robustness/fragility of the main results to reasonable perturbations of the assumptions and statistical methods used; for example, examination of the impact of (1) including/excluding questionable data, (2) representing PriCARE exposure in terms of the number of attended PriCARE sessions (dose) instead of intent-to-treat allocation, (3) excluding caregivers assigned to PriCARE who attended ≤ 3 of the 6 sessions, (4) using a generalized logistic mixed-effects model, (5) using a generalized log-linear model for the binary outcome (CPS investigation yes/no) or for the dyad’s number of CPS investigations, (6) using a Cox proportional hazards model for time to first CPS investigation. The sensitivity analyses will only be used to guide our level trust in the main results and guide wording of our conclusion statements.

#### Sensitivity analyses objective 2

To guide our level of trust in the main results about effectiveness, sensitivity analyses will be used to evaluate the robustness/fragility of the main results to reasonable perturbations of the assumptions and statistical methods used; for example, examination of the impact of (1) including/excluding questionable data, (2) using a constrained longitudinal generalized linear model which would be able to include all subjects—even those without follow-up data at 6–8 months, (3) representing PriCARE exposure in terms of the number of attended PriCARE sessions (dose) instead of intent-to-treat allocation, (4) excluding caregivers assigned to PriCARE who attended ≤ 3 of 6 sessions.

### Methods in analysis to handle protocol non-adherence and any statistical methods to handle missing data {20c}

For the main analysis, we will assume that any missing data values were caused by ignorable mechanisms [97]. Additional sensitivity analyses will be used as described in {20b}.

### Plans to give access to the full protocol, participant level-data and statistical code {31c}

This publication grants public access to a concise summary of the full protocol. De-identified individual participant data collected during the trial will be made available beginning 36 months following the article (primary objective) publication and ending 60 months following article publication. Selected code for statistical computations may be available upon request.

## Oversight and monitoring

### Composition of the coordinating center and trial steering committee {5d}

Daily monitoring of the study locally will be done by the research coordinators and the two PIs at their respective institutions. The entire research team will meet at least every other week during subject enrollment.

### Composition of the data monitoring committee, its role and reporting structure {21a}

Study oversight will be under the direction of a Data and Safety Monitoring Board (DSMB) composed of a formal independent board of experts, including investigators with backgrounds in psychology, child welfare research, clinical trials of pediatric behavioral health interventions, and biostatistics.

### Adverse event reporting and harms {22}

The Chair of the DSMB will serve as the safety officer and will be the contact person for serious adverse event reporting. The PIs will be responsible for notifying the DSMB chair and IRB of any serious adverse events when they happen, in accordance with IRB regulations.

### Frequency and plans for auditing trial conduct {23}

Meetings of the DSMB will be held at least two times a year at the call of the Chair. An emergency meeting of the DSMB may be called at any time by the Chair should participant safety questions or other unanticipated problems arise.

### Plans for communicating important protocol amendments to relevant parties (e.g. trial participants, ethical committees) {25}

The date of each amendment, accompanied by a description of the change and the rationale will be submitted to all overseeing safety boards prior to implementing.

### Dissemination plans {31a}

The results of this trial will be published in peer-reviewed journals as well as at national conferences. Results will also be disseminated at the participating health systems through presentations at primary care practitioner-focused conferences, participation in primary care podcast series, and other venues.

## Discussion

In spite of the high prevalence and documented adverse health consequences of CM, there is a paucity of research on preventive interventions [98]. The promotion of clinical research that aims to provide empirical information to support preventive CM interventions was stated as a priority 25 years ago by the National Research Council (NRC), a statement recently reaffirmed by the World Health Organization [99], the NAM, and the NRC [16]. In particular, research on effective CM prevention interventions delivered in primary care settings is lacking. The primary care setting is an ideal venue to engage families in prevention, as it is the only place where most (>95%) US preschool-aged children visit at least once/year [100]. Yet, in 2018, the US Preventive Services Task Force concluded that there was insufficient evidence to assess the balance of benefits and harms of primary care interventions to prevent CM [101, 102]. Thus, there is a critical need for research informing the development and implementation of CM prevention programs in primary care.

In addition to being the largest CM prevention trial with individual randomization, this study addresses three of the key research priorities identified by the NAM and NRC in their report on “New Directions in Child Abuse and Neglect Research.” The first priority is developing and testing new programs for underserved children and families. PriCARE/*CARIÑO* was developed with input from and evaluated in underserved families. The existing evidence base favoring its efficacy is strong [38–41]. Three RCTs and one pre/post pilot have demonstrated improvements in key proximal outcomes, including child behavior problems, harsh parenting, and parent stress scores. Our proposed study is well-designed to evaluate the impact of this promising intervention on CM prevention. Furthermore, our study population is drawn from diverse sites, including clinics in which PriCARE/*CARIÑO* is well established, new sites, academic and private clinics, as well as urban and suburban locations, which will contribute to the generalizability of the results.

The second priority is identifying the most effective ways to implement and sustain evidence-based programs in primary care settings. To achieve the public health impact of reducing CM, PriCARE/*CARIÑO* must not only be effective but must also be widely implemented and sustained [103]. Evidence-based interventions implemented without effective screening, referral, and enrollment processes will make little impact on improving the lives of children and families on a broad scale [103, 104]. This study will generate data critical to conducting a future study of implementation strategies to inform scale-up of parenting interventions in primary care settings. Our study design provides an early opportunity to learn about implementation in diverse practice settings. There is a clear need to understand key PriCARE/*CARIÑO* implementation determinants (barriers and facilitators) and leverage that data to develop strategies to promote the implementation, scale-up, and sustainment of PriCARE/*CARIÑO*.

The third priority our study addresses is the investigation of the longitudinal impacts of CM prevention, which can be challenging and costly to initiate. By including consent for the longitudinal data collection on a cohort of nearly 2000 caregiver-child dyads, this study will lay the foundation for future longitudinal studies of the long-term impact of the program on families. This proposed work will serve as the basis for future longitudinal studies evaluating the continued impact of the intervention on CM rates as well as on additional distal outcomes such as academic performance and mental health diagnoses. Furthermore, by testing multiple proximal outcomes in addition to the more distal outcome of CM and contextualizing the results with qualitative data [105], this study will help to elucidate the mechanisms of change and advance the science of CM prevention.

If successful, results from this study will be used to seek funding from state agencies and payers to sustain PriCARE/*CARIÑO* in primary care, while continuing to refine and study implementation and dissemination strategies, with the ultimate goal of achieving levels of exposure sufficient for breaking the intergenerational transmission of CM.

### Trial status

Enrollment started May 18, 2022, on and the estimated end date of enrollment is January 1, 2026. This trial was registered on ClinicalTrials.gov (NCT05233150) on February 1, 2022, prior to enrolling subjects.

## Data Availability

Both Principal Investigators and the statistical analysis team will have access to the final cleaned de-identified dataset.

## Abbreviations

BCAP: Brief Child Abuse Potential Inventory
CM: Child maltreatment
CPS: Child protective services
CTS-PC: Conflict Tactics Scales, Parent-Child version
DPICS: Dyadic Parent-Child Interaction Coding System
DSMB: Data and Safety Monitoring Board
ECBI: Eyberg Child Behavior Inventory
EMR: Electronic medical record
GEE: Generalized estimating equation
NAM: National Academy of Medicine
NRC: National Research Council
PS: Parenting Scale
PSI-SF: Parenting Stress Index-Short Form
REDCap: Research Data Capture
SES: Socioeconomic status
TDF: Theoretical Domains Framework
